# Meat and Fish Freshness Assessment by a Portable and Simplified Electronic Nose System (Mastersense)

**DOI:** 10.3390/s19143225

**Published:** 2019-07-22

**Authors:** Silvia Grassi, Simona Benedetti, Matteo Opizzio, Elia di Nardo, Susanna Buratti

**Affiliations:** 1 Department of Food, Environmental, and Nutritional Sciences (DeFENS), Università degli Studi di Milano, via G. Celoria 2, 20133 Milan, Italy; 2 Senior S.r.l., via Molino 2, 21052 Busto Arsizio, Italy

**Keywords:** electronic nose, food quality, MOS sensors, K-Nearest Neighbours’ algorithm (K-NN), Partial Least Square-Discriminant Analysis (PLS-DA)

## Abstract

The evaluation of meat and fish quality is crucial to ensure that products are safe and meet the consumers’ expectation. The present work aims at developing a new low-cost, portable, and simplified electronic nose system, named Mastersense, to assess meat and fish freshness. Four metal oxide semiconductor sensors were selected by principal component analysis and were inserted in an “ad hoc” designed measuring chamber. The Mastersense system was used to test beef and poultry slices, and plaice and salmon fillets during their shelf life at 4 °C, from the day of packaging and beyond the expiration date. The same samples were tested for Total Viable Count, and the microbial results were used to define freshness classes to develop classification models by the K-Nearest Neighbours’ algorithm and Partial Least Square–Discriminant Analysis. All the obtained models gave global sensitivity and specificity with prediction higher than 83.3% and 84.0%, respectively. Moreover, a McNemar’s test was performed to compare the prediction ability of the two classification algorithms, which resulted in comparable values (*p* > 0.05). Thus, the Mastersense prototype implemented with the K-Nearest Neighbours’ model is considered the most convenient strategy to assess meat and fish freshness.

## 1. Introduction

The evaluation of meat and fish quality is of primary importance since producers and retailers have to ensure that products are safe and able to meet the consumers’ expectation.

Meat and fish are highly perishable due to the influence of many post-mortem factors, so their loss of quality (safety, nutritional, and sensory properties) is very rapid. The shelf life of these products can be defined as the time occurring between production and spoilage, caused by biochemical reactions and microbiological activity [1].

The commonly used methods to evaluate the spoilage status of meat and fish and the changes associated with the loss of quality include microbiological determination and sensory analysis. The Total Viable Count (TVC) of bacteria is recognised as an indicator of the safety evaluation of meat products able to give an overall scenario of microbiological spoilage [2]. However, even if there are specific limits for pathogenic bacteria presence [3], no European limit exists for TVC in meat and fish product. In Italy, at the regional level, some attempts have been made. The Piedmont region defined safety levels based on TVC for meat and fish products based on three classes of freshness [4]. Sensory analysis is not always applicable, even though there are quality assessment methods for fish evaluation based on it [5]. Microbiological and sensory analysis have some drawbacks since they are destructive, expensive, and require skilled personnel; moreover, microbial analysis by plate count requires long incubation periods, thus leading to results after 2–3 days.

Some chemical compounds (alcohols, H_2_S, acetate, methyl ethyl ketone, dimethyl sulphide, etc.) may be considered as spoilage indicators, but their quantitative determination involves laborious extraction and analytical procedures; furthermore, limited information is provided [6].

Over the last few decades, important efforts have been made in order to develop non-destructive sensing methods applicable in situ or on-line to assess the freshness and the quality of food [7,8].

Nowadays, the electronic nose (e-nose) is commonly used in numerous fields including environmental, medical, and pharmaceutical [9]; in the food industry, this device has proven to be very effective for a number of purposes such as quality control, process monitoring, freshness evaluation, shelf life investigation, and authenticity assessment [10,11,12].

In the last twenty years, many studies have been published concerning the use of the electronic nose to evaluate the shelf life and the quality of meat and fish products by measuring the volatile compounds (aldehydes, ketones, esters, sulphur, and ammonia compounds) produced during storage by microbial growth and biochemical reactions [13,14,15]. In many applications, MOS (Metal Oxide Semiconductors) sensors have been applied since they are readily available on the market and, at the same time, suitable for use due to their robustness and durability, as well as for their rapid response and good sensitivity to volatile compounds [16]. In general, MOS sensors are highly sensitive to aldehydes, alcohols and ketones; furthermore, by adjusting their working temperature, it is possible to increase the sensitivity and the selectivity towards other compounds such as aromatic molecules, terpenes, and organic acids [17].

In [18], the authors assessed the shelf life of fresh pork meat stored under refrigeration in aerobic conditions by an electronic nose pocket device, the Food Sniffer^®^ (ARS.LAB Inc., Redwood City, CA, USA). The device is controlled via a Smartphone application and detects temperature, humidity, and volatile compounds. The Food Sniffer^®^ is an easy-to-use device, which is meant for consumer use in specific and predefined applications.

Multivariate data analysis applied to e-nose results proved to be effective to extract relevant information from the sensor signals improving pattern recognition [19]. A recent paper [20] reviewed the applications of sensing techniques, including e-nose, and the chemometric approaches used for fish freshness evaluation. The authors reported a large use of Support Vector Machine (SVM), Artificial Neural Network (ANN), and Partial Least Square-Discriminant Analysis (PLS-DA) for the modelling of fish freshness. Even if less is applied, K-Nearest Neighbours’ algorithm (K-NN) demonstrated to be more reliable than SVM and ANN in classifying meat and fish quality when developing an odour sensing system [21].

In this context, the aim of the present work was the development of a new low-cost, portable, and simplified e-nose system, named Mastersense, operating with four MOS sensors.

The four MOS sensors were selected by Principal Component Analysis (PCA), then two different classification methods, K-NN and PLS-DA were developed to classify the freshness of samples using e-nose information, in line with previous authors [22].

The combination of the designed electronic nose and the classification models developed will provide a friendly system for reliable, sensible, and specific classification of the meat/fish samples in three freshness categories. A strong point of the device is the versatility of the acquisition system implemented for off-line, in-line, and on-line applications. The commercial device could be adapted to specific needs of producers and retailers who will be able to develop ad hoc models by collecting their own data, thus enlarging the field of applicability to different meat species and cuts, as well as to different fish species. Thus, the portable and simplified electronic nose system will be a valuable solution to inform producers, retailers, and consumers on products safety and quality.

## 2. Materials and Methods

### 2.1. Electronic Nose System Development

The development of the e-nose system has involved the design and the implementation of the measuring chamber where the four MOS sensors are located, the electronic sensor boards, the electronic motherboard and the pump, and the implementation of the acquisition system.

#### 2.1.1. Measuring Chamber

**Figure 1:**
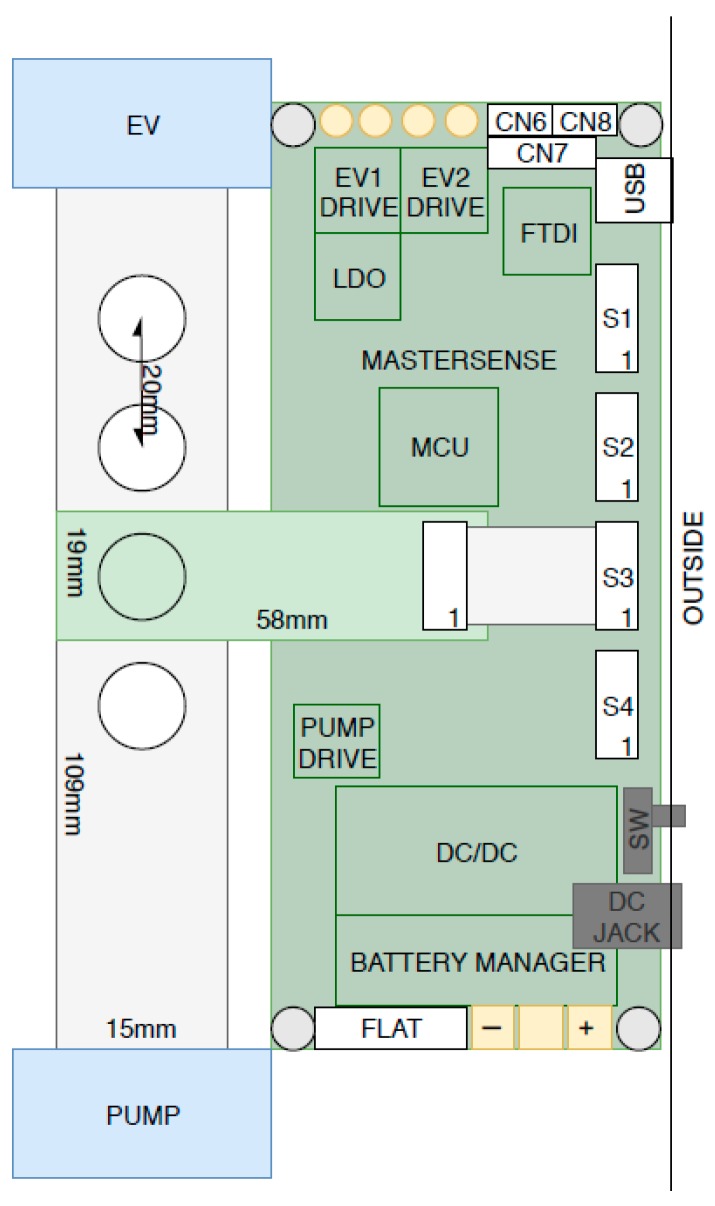
Layout of the measurement chamber: EV = solenoid valve; EV1 drive = control driver for solenoid valve; EV2 drive = driver for additional solenoid valve; CN6-CN7-CN8 = connectors dedicated for settings; FTDI = USB to Serial converter; USB = USB port; S1-S2-S3-S4 = connectors for the four sensor’s board; MCU = microcontroller; PUMP DRIVE = pump controller; DC/DC = DC/DC 12 V out converter for battery charge management; FLAT = Connector used for debugging; PUMP = brushless pump (model KNF NMP 03 KPDCB-1, 3.3 Volt) for continuous 24 h operation; SW = ON/OFF switch; DC JACK = power supply input (DC 15–36 V).

The layout of the Mastersense measuring chamber (150 × 100 mm) with all its components is presented in Figure 1.

Starting from the left of Figure 1, it is possible to see a pump drawing the filtered air (during purging time), the sample headspace (during the sampling time), and the solenoid valve (EV) controlling the flow rate; in-between there are four holes, drilled at regular intervals, for sensor positioning. An array of four sensors (GGS 8530, GGS 5430, GGS 2530, and GGS 10530) selected out of ten tested MOS sensors (Table 1), produced by UST Umweltsensortechnik GmbH (Geschwenda Germany), has been inserted in the measuring chamber [23].

**Figure 2:**
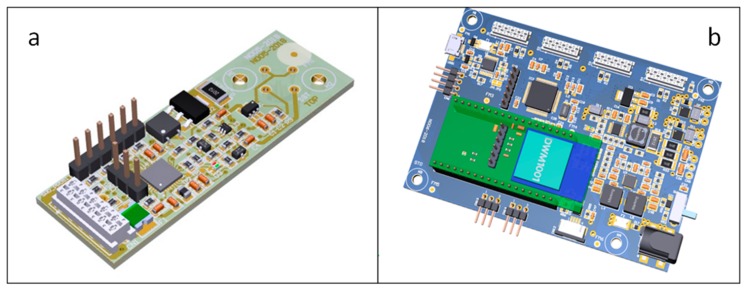
Sensor control board (**a**) and motherboard (**b**).

Each sensor was located on a special board (60 × 20 mm) equipped with the electronics required to regulate its temperature and to perform the measurements (Figure 2a). The electronic boards were fixed on the measuring chamber so that each sensor, located in a specific hole, was connected to the motherboard by a “strip” carrying the communication signals and the power supply.

The pump consisted of a brushless motor whose electronic control (PUMP DRIVE) has been developed “ad hoc” and positioned on the motherboard (Figure 2b) that controlled all the components: the solenoid valve, the pump, the power supply (including the battery), and the sensor boards.

#### 2.1.2. Acquisition System

The acquisition system has been implemented by considering three possible applications:

- Off-line mode: the control software and the pattern recognition system are installed on a PC sending commands to the e-nose and receiving data; the connection could be wireless (WIFI) or wired (Ethernet).
- In-line: the control software and the pattern recognition system are included directly in the e-nose firmware. The e-nose sends the result of pattern recognition to PC to control the production line.
- On-line mode: different e-noses could be wireless connected to a cloud platform and send the collected data to a server via an internet connection. The control software and the pattern recognition system are in the cloud. The users will be able to access the data by a smart client application for computer, or by mobile applications for tablet and smartphone.

All data collected by on-line or off-line mode could be stored on the cloud platform ad hoc developed for the Mastersense.

### 2.2. System Testing, Data Acquisition, and Laboration

The testing phase involved the selection and preparation of samples, the data acquisition by headspace sampling, the data analysis, and the development of predictive models using multivariate statistical methods.

#### 2.2.1. Samples and Sample Preparation

Meat and fish samples were directly purchased from local retailers, in particular, beef (scottona, aka meat of young heifer not older than 15–16 months) slices (250 g–300 g) from the *long digital extensor* and *long digital flexor* muscles, poultry slices (400 g), European plaice fillets (250 g), and salmon fillets (250 g), packaged in polystyrene trays wrapped with a transparent laser microperforated film permeable to oxygen, were considered. Each tray corresponds to an independent sample, i.e., an animal, for chicken and fish, whereas for beef at each sampling time were selected two trays containing slices from different beef loins.

Analyses were carried out at set times during sample storage, from the day of packaging (0) or from the day after packaging (1), beyond the expiration date as indicated on the product label and up to the safety and quality threshold of 10^7^–10^8^ CFU/g reported in many studies [24] and assessed by the TVC (as reported in Section 2.2.3). During the storage period, samples were kept at 4 °C, representing the typical meat and fish storage temperature in supermarkets and grocery stores. Measurements were replicated on several series and, at each storage time, analyses were performed, TVC, and electronic nose analyses in duplicate/triplicate on two packages. Details of the analysed series and storage times are shown in Table 2. For each series of products (different purchasing batch), the number of replicates able to guarantee the analysis of two trays for the investigated sampling times was considered for a total of 80 samples for beef, 88 samples for poultry, 72 samples for salmon, and 63 samples for European plaice.

#### 2.2.2. E-nose Analysis

Meat and fish samples (50 g) were placed in 250 mL airtight glass jars fitted with a pierceable Silicon/Teflon disk in the cap. After one hour of headspace equilibration at room temperature, the measurement started by pumping the sample headspace over the sensor surfaces for 60 s (sampling time) during which the sensor responses were recorded; the sample headspace was withdrawn to a flow rate of 400 mL/min, and the sampling frequency was 1 Hz. After sample analysis, sensors were purged for 180 s with filtered air (purging time), then, prior to the next sample injection, the sensor baselines were re-established for 5 s. The sensor response corresponded to the fractional value obtained by subtracting the resistance signal of the baseline (R0-ohm) from the resistance signal of the sensors (R-ohm) and dividing by the resistance signal of the baseline (R0), thus providing a dimensionless, normalised response. Sensor responses were acquired at 50 s of sampling and statistically elaborated. The sensor selection and the time required for sampling, purging, and data acquisition were determined through preliminary tests.

#### 2.2.3. Microbial Analysis

The TVC was carried out as reported in the UNI EN ISO 4833-1: 2013 [25]. The International Organization of Standardization (ISO) standard specifies a horizontal method for the enumeration of microorganisms that are able to grow and form colonies in a solid medium after aerobic incubation at 30 °C. The method is applicable to samples in the area of food and feeds production and handling. An overview of the TVC results is reported as the mean value (CFU/g of sample) for each sampling time, i.e., the average of the replicates and trays.

#### 2.2.4. Data Analysis and Predictive Model Development

Microbiological results were subjected to one-way analysis of variance (ANOVA) considering the storage times as factors and microbial plate counts as dependent variables. When a significant effect (*p* < 0.05) was found from ANOVA, a multiple comparison post-hoc test, Fisher’s Least Significant Difference (LSD), was applied at a 95% confidence level. The storage times resulting were not significantly different by the LSD test (*p* > 0.05) were considered as belonging to the same group.

To select the four out of 10 sensors to be implemented in the Mastersense system, e-nose data from 10-MOS sensor-based e-nose datasets were transformed by column autoscaling and then explored by PCA. PCA is an unsupervised exploratory procedure that allows to visualising, in a reduced space, the relationships between objects and variables, thanks to graphical outputs (i.e., score plot, loading plot and bi-plot) [26]. In our case, the inspection of the biplot allowed to understand the variables influence in the object dispersion and the selection of the four most informative ones, i.e., the four sensors to be implemented in the portable system. PCAs were than performed on the data collected from the 4-MOS sensor-based e-nose devices to confirm the distribution of the samples according to freshness level was confirmed.

In order to develop classification models, two classification approaches were used: 4-MOS sensor-based e-nose: K-NN and PLS-DA. K-NN is a simple non-linear classification approach based on the Euclidean distance and not requiring any assumptions on the underlying data distribution. In detail, K-NN predicts the class membership of a sample on the basis of the class of the K nearest sample(s) in the multidimensional space; in this work a K value of 3 was applied, which should be considered a good compromise as a small value of K allows high influence of noise and a large value makes the model computation expensive for real applications [27].

PLS-DA applies the PLS regression bases to a *Y* dummy and completes a rotation of the projection to latent variables searching for the maximum separation among classes. The *Y* dummy matrix is constructed so that it has a number of columns equal to the number of classes, and each column is filled with ones and zeros, being one when the object belongs to the considered class and zero otherwise [28].

Before the development of the classification models, each dataset was split by the Kennard–Stone algorithm [29] into a training and a test set composed of about 70% and 30% of the original samples, respectively: beef (55 samples for training, 25 sample for test), poultry (58 samples for training, 30 sample for test), plaice (43 samples for training, 20 sample for test), and salmon (47 samples for training, 25 sample for test). The training dataset was used for the classification rule development and the internal validation by cross-validation with five cancellation groups, whereas the test set was used to test the model’s performance in prediction.

The predefined classes were used as a-priori information (Y) to build classification models able to predict meat and fish freshness based on the e-nose data collected from the selected sensors (X). The applied classifiers (KNN and PLS-DA) were evaluated by two metrics: sensitivity and specificity computed as reported in Table 3 on the bases of four-factor: True Positive (TP), False Positive (FP), True Negative (TN) and False Negative (FN). Sensitivity expresses the model capability to correctly recognize samples belonging to the considered class; whereas specificity describes the model capability to reject samples belonging to all the other classes correctly. Sensitivity and specificity can assume values between 0 and 1, where 1 indicates perfect classification and 0 no correct prediction. For PLS-DA models, ROC (Receiver Operating Characteristic) curves were used to assess and optimize the specificity and sensitivity of each class with different thresholds.

Furthermore, weighted sensitivity and specificity were calculated for each model by the equation:

$${\bar{X}}_{weighted}=\;\frac{\left(m_{1}f_{1}+m_{2}f_{2}+m_{3}f_{3}+m_{4}f_{4}\right)}{f_{1}+f_{2}+f_{3}+f_{4}}$$

where m_i_ is the SENS or SPEC for the i-class and f_i_ is the i-class numerosity.

Classification performances were compared by a particular case of Fisher’s sign test, named McNemar’s test, that verifies if two models have the same error rate [30]. For further details about the statistical test, the reader is referred to Grassi et al. (2018) [31].

All the data analyses were performed under Matlab environment (R2017b, The Mathworks, Inc., Natick, MA, USA) eventually using the PLS toolbox (ver. 8.5, Eigenvector Research, Inc., Manson, WA, USA) software package.

## 3. Results and Discussion

### 3.1. Sensor Selection

In order to select four MOS sensors to be inserted into the portable and simplified e-nose system, a device embedded with an array of 10 MOS sensors was used for the preliminary experiments. In Table 2, the details of the tested sensors purchased from UST Umweltsensortechnik GmbH (Germany) are reported.

**Figure 3:**
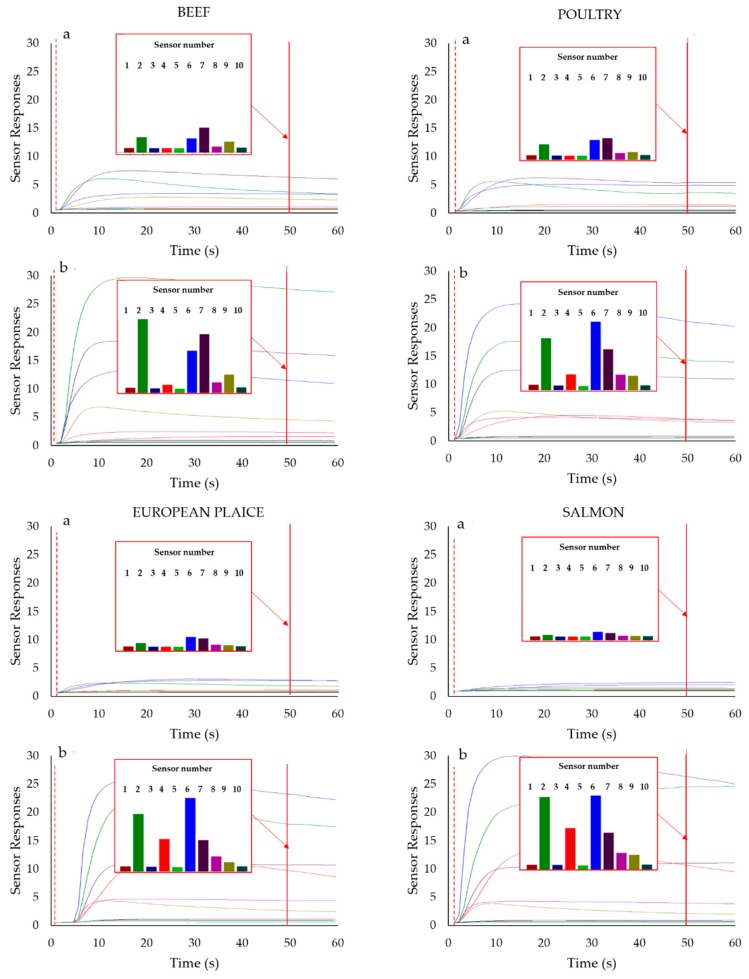
Metal oxide semiconductor sensor responses for meat and fish samples collected the first day of sampling (**a**) and at the expiration date of the product (**b**). The histograms represent the 10 sensor responses after 50 s of sampling. Each bar colour corresponds to a sensor.

Figure 3 shows the sensor responses collected during the sampling phase on meat (beef and poultry) and fish (salmon and plaice) samples on the first day of analysis (a) and the day corresponding to the expiration date of the product (b). The x-axis represents the sampling time while the sensor response is reported on the y-axis.

As can be seen in Figure 3, during sampling, the sensor responses gradually increased, reaching a plateau after 25–30 s. From the histograms, representing the responses collected after 50 s of sampling, it is possible to notice that the sensor responses are very low for the fresh products (first day of analysis) while, at the end of their commercial life (seven days for beef and poultry; three days for plaice, and four days for salmon), the responses of S2, S6, and S7 sensors were significantly increased.

A further elaboration of the collected data by PCA (figures not shown) allowed the selection of the sensors more suitable to follow the evolution of the products’ headspace during storage. In particular, the S1 (GGS 8530) sensor has been selected for its ability to discriminate fresh meat and fish samples, while S2 (GGS 5430), and S6 (GGS 2530), S7 (GGS 10530) sensors characterise samples at the end of their commercial life; moreover, they are those increasing more significantly their response signals during the storage of the samples. In detail, GGS 8530 has the ability to detect hydrogen and aliphatic compounds working to a temperature range of 150–260 °C, GGS 5430 is sensitive to ammonia compounds (working temperature 220–350 °C), GGS 2530 has high sensitivity and low specificity (working temperature 250–300 °C); GGS 10,530 is sensitive to sulpher compounds (working temperature 360–450 °C).

### 3.2. Microbiological Analysis and Class Identification

In this work, three classes of freshness, corresponding to a traffic light system, were established. In order to define the three classes, the results of the TVC collected for all products (beef, poultry, salmon, and plaice) were submitted to one-way ANOVA and to the LSD test to identify groupings between samples. In Table 4 and Table 5, the results of ANOVA and the LSD test are reported.

Considering beef samples (Table 4), the LSD test has identified the presence of five groups (a; b; c; d; e); since the number of groups to be formed was equal to three, a further grouping was applied according to the following criterion:

Unspoiled samples: ANOVA group “a”; microbial count < 10^6^
Acceptable samples: ANOVA group “b”; 10^6^ < microbial count < 10^7^
Spoiled Samples: ANOVA “c; d; e”; microbial count > 10^7^

The same procedure was performed also for poultry, salmon, and plaice samples and the final groupings are reported in Table 4 and Table 5 (columns Group).

Being the identified groups acceptable from a scientific point of view [21,22,23,24] and in accordance with the guide lines defined by the Piedmont region for safety levels based on TVC for meat and fish products [4], they were upgraded to the rank of classes: green class-unspoiled; yellow class-acceptable, red class-spoiled; the predefined classes are summarised in Table 6.

### 3.3. PCA of Electronic Nose Data

**Figure 4:**
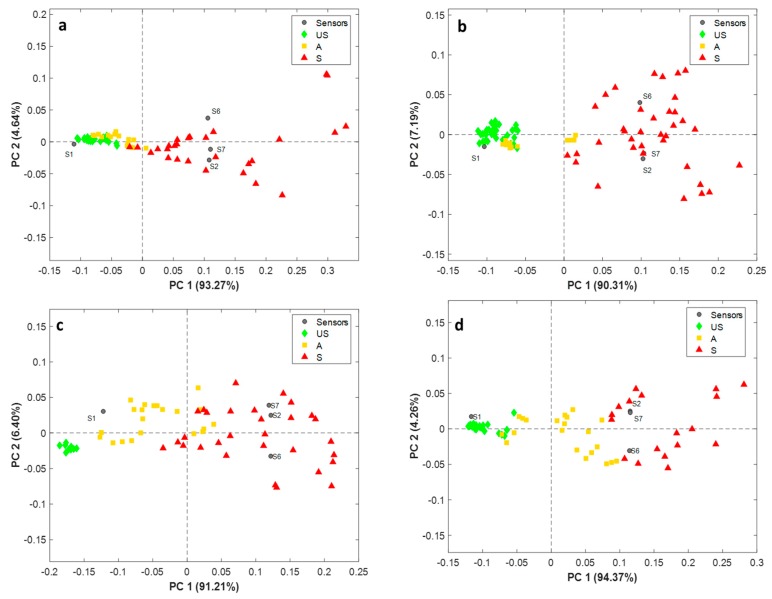
Principal Component Analysis-biplots of e-nose data collected on beef (**a**) poultry (**b**) plaice (**c**) and salmon (**d**) samples classified as unspoiled (US)-green; acceptable (**A**)-yellow, spoiled (**S**)-red.

In Figure 4, the PCA-biplots of the four sensor responses collected at 50 s of sampling on all the analysed samples are reported: beef (**a**), poultry (**b**), plaice (**c**), and salmon (**d**). The samples are coloured on the basis of the previously defined classes: green-unspoiled; yellow-acceptable; and red-spoiled. As can be seen, almost all the samples cluster according to the three predefined classes in the PC1 vs. PC2 plane; moreover, in the case of beef (Figure 4a) and poultry (Figure 4b), unspoiled (green) and acceptable (yellow) samples assume similar PC1 scores, whereas spoiled (red) samples are characterised by higher PC1 scores and are more dispersed along PC1 and PC2. However, for both products, the overlap between classes appears to be very limited, and the unspoiled samples (green) are never confused with the samples a-priori defined as spoiled (red). Similar considerations can be drawn for plaice (Figure 4c) and salmon (Figure 4d) fillets; thus, demonstrating the synergic effect of the selected sensors to discriminate the products suitable for consumption from those that are acceptable and above all, not suitable.

### 3.4. Classification Model Development (KNN and PLS-DA)

#### 3.4.1. K-NN Models

K-NN models (K = 3) were developed for all products and in Table 7, the metrics calculated for each model in the calibration, cross-validation, and prediction are reported.

Concerning beef, in the cross-validation phase, sensitivity of 0.94, 0.77, 0.75 was reached for unspoiled, acceptable, and spoiled classes, respectively. A few cases of misclassification were reported in cross-validation, leading to a specificity 0.86 for the unspoiled class, 0.90 for the acceptable class, and 1.00 for the spoiled class. Good performances were obtained in prediction. A few acceptable samples were misclassified; this result is understandable as this class is characterised by an intermediate freshness level, which could be more easily confused with the neighbour classes.

Concerning poultry, model cross-validation gave good sensitivity values, i.e., 0.92, 0.70, and 0.91 for unspoiled, acceptable, and spoiled classes, respectively (Table 7). Even better, the specificity calculated for the cross-validation was 0.86, 0.90, and 1.00, respectively, for the three classes. The poultry K-NN model did not perform as good as the beef model in prediction, mainly because the four samples in the spoiled class and one sample in the unspoiled class were predicted as acceptable, leading to poor sensitivity of these classes (0.84 and 0.66, respectively) and reduced specificity for the acceptable class (0.82).

European plaice K-NN model gave robust results in the cross-validation with sensitivity ranging from 1.00 to 0.71 and specificity from 0.97 to 0.88 (Table 7). Optimal performances were obtained in prediction. Indeed, for almost all the classes, sensitivity and specificity were 1.00. However, two out of eight samples a-priori identified as acceptable were erroneously predicted as spoiled. A type II error, i.e., rejecting a sample which is then classified as inadequate, confirms the real-life feasibility of the model which will not bring any safety risk.

With regard to the internal validation of the salmon model, good sensitivity was reached, and was 0.93, 0.85, and 0.80 for the three classes (Table 7). The specificity in the cross-validation was quite high, i.e., 0.89, 0.91, and 1.00, respectively, for the unspoiled, acceptable, and spoiled class. In the prediction, high performance was obtained (>0.88), except for the sensitivity of the unspoiled class (0.75).

Despite being one of the simplest approaches for classification, K-NN has shown to perform quite good in the tested application (*p*-values in prediction > 0.80), leading to models with sensitivity in the prediction between 0.83 and 0.92, and specificity between 0.88 and 0.99.

#### 3.4.2. PLS-DA Models

PLS-DA models were developed to test if a more complex approach, based on the optimisation of covariance between X and Y, could lead to better classification performance. The model performance in terms of sensitivity and specificity in calibration, cross-validation, and prediction are reported in Table 8. ROC curves were used to assess and optimise the specificity and sensitivity of each class with different thresholds, being 0.41, 0.34, 0.44, and 0.32 for beef, poultry, plaice, and salmon, respectively.

The internal validation (cross-validation) of the beef model performed well for the unspoiled and the spoiled classes reaching sensitivity above 0.82 and specificity higher than 0.80; however, the sensitivity of the acceptable class was quite low (0.54). The same considerations are valid for the external validation (aka. prediction) (Table 8). Similarly, Linear Discriminant Analysis (LDA) and Quadratic Discriminant Analysis (QDA) models were developed to discriminate unspoiled for spoiled beef (cut off 6 log_10_ CFU/g of TVC) [32] reaching overall classification accuracy in cross-validation up to 0.89 and 0.93 for linear and quadratic models, respectively. In the literature, more complex nonlinear approaches were also investigated: Artificial Neural Network (ANN) classification reached an accuracy in the cross-validation ranging from 83 to 100% [33]; by Support Vector Machine, a correct classification rate of 98.8% has been obtained [13].

Concerning poultry, the cross-validation model performed similarly to the K-NN algorithm with sensitivity between 0.70 and 0.92 and specificity ranging from 0.85 to 1.00. Better performances were recorded in prediction, achieving both a sensitivity and specificity higher than 0.91. (Table 8).

The European plaice PLS-DA model gave robust results in cross-validation with sensitivity ranging from 0.78 to 1.00 and specificity from 0.86 to 1.00 (Table 8). Optimal performances were obtained in prediction.

The PLS-DA model developed for the salmon samples resulted in less robust results than the corresponding K-NN classifier. In particular, it failed in classifying correctly the samples belonging to the spoiled class by cross-validation (sensitivity of 0.40). However, the model performance in prediction was quite high. A few studies present in literature discuss e-nose implementation for quality assessment of fish products. Among them, PLS regression models have been developed for microbial count prediction, obtaining high R^2^ (0.96) and low error (0.32 log_10_ CFU/g) in prediction [34].

#### 3.4.3. Classification Results Comparison

To better compare the K-NN and PLS-DA performances, the global sensitivity and specificity reached in prediction are reported in Table 9. Predicted sensitivity of PLS-DA models resulted in equal or higher than the percentages reached by K-NN models, whereas the specificity percentages did not follow a clear trend. To objectively compare the models’ quality, a McNemar’s test was performed on the predicted classes of each model. With the McNemar’s test, the accuracy for predicting the a-priori defined classes (Y) of K-NN and PLS-DA models has been evaluated. In all cases the classification loss of each model in prediction was quite low. Indeed, the K-NN error ranged from 0.167 to 0.080; whereas, the PLS-DA error was slightly lower (0.040–0.100) (Table 9). The misclassification error is complementary to the model accuracy; thus, the overall accuracy (%) of the K-NN models ranged from 83.3% to 92%, whereas for PLS-DA models it was between 96% and 90%.

Notwithstanding the classification loss differences, the models developed by the two different algorithms were comparable (*p* > 0.05) in terms of prediction capabilities.

## 4. Conclusions

The new portable and simplified 4-MOS sensor-based e-nose system, named Mastersense implemented with a classification algorithm (K-NN) developed in the present study was able to correctly classify meat (beef and poultry) and fish (plaice and salmon) samples into three freshness classes defined by TVC (green-unspoiled, yellow-acceptable, red-spoiled).

The classification results obtained in prediction demonstrated the ability of Mastersense to correctly distinguish and classify meat and fish samples on the basis of their freshness. Even though some samples belonging to intermediate freshness level were misclassified, none of the samples with risky microbial concentration (red) was classified as acceptable for consumption. This reveals that the model can be safely used for real applications.

As a future perspective, to overcome the weakness of the developed system and to adapt the device to specific needs of producers and retailers, reduced sampling times could be experimented and ad hoc classification models developed, thus enlarging the device applicability.

## Figures and Tables

**Table 1:** Tested sensors and related specifications.

Sensor Name	Sensor Type	Sensor Sensitivity
S1	GGS 8530	Sensor for the detection of C_2_H_5_OH, with low cross-sensitivity to CH_4_, CO and H_2_
S2	GGS 5430	Sensor especially sensitive to NO_2_ (nitrogen dioxide) and O_3_ (ozone)
S3	GGS 7330	Sensor for the detection of NO_X_
S4	GGS 6530	Sensor for the detection of H_2_, with low cross-sensitivity to CH_4_, CO and alcohol
S5	GGS 3530	Sensor for the detection of hydrocarbonates, optimal for C1 C8-hydrocarbonate
S6	GGS 2530	Sensor with a high sensitivity to CO, H_2_ and C_2_H_5_OH and a low cross-sensitivity to CH_4_
S7	GGS10530	Sensor for the detection of selected VOCs in the trace range
S8	GGS 1530	Universal sensor with many applications
S9	GGS 4430	Sensor for NH_3_ (ammonia), with low cross-sensitivity to CH_4_, CO and H_2_
S10	GGS 1430	Universal sensor with many applications

**Table 2:** Experimental design.

	BEEF			POULTRY			SALMON		EUROPEAN PLAICE	
	Series 1	Series 2	Series 3	Series 1	Series 2	Series 3	Series 1	Series 2	Series 1	Series 2
**Times (days) from the day of packaging**	0	0	0	-	-	0	-	-	-	-
	1	-	-	1	1	1	1	1	1	1
	2	-	-	2	-	2	2	2	2	2
	3	3	3	3	-	3	3	3	3 *	3 *
	4	4	4	4	4	-	4 *	4 *	4	4
	-	5	5	-	5	-	5	5	5	5
	-	6	-	-	6	-	-	-	-	-
	7 *	7 *	-	7 *	7 *	-	-	-	-	-
	9	-	-	8	8	-	8	8	-	-
	8	-	-	9	-	-	-	-	-	-
	10	10	-	10	-	-	-	-	-	-
	-	-	-	-	11	-	-	-	-	-

* Expiration date indicated on the label

**Table 3:** Generic confusion matrix identifying true positive (TP), false positive (FP), true negative (TN), false negative (FN), and the reference equation to calculate sensitivity (SENS) and specificity (SPEC).

		A-Priori		
		Class 1	Class 2	
**Predicted**	**Class 1**	TP	FP	$SENS=\frac{TP}{\left(TP+FN\right)}$
	**Class 2**	FN	VN	$SPEC=\frac{TN}{\left(TN+FP\right)}$

**Table 4:** One-way ANOVA and Least Significant Difference (LSD) test applied on microbiological data (Total Viable Count, CFU/g of product) collected on beef and poultry samples.

BEEF					POULTRY				
Series	Time (Days)	CFU/g	ANOVA LSD *	Group	Series	Time (Days)	CFU/g	ANOVA LSD *	Group
1	0	1.60 × 10^4^	a	Unspoiled	1	1	3.40 × 10^4^	ab	Unspoiled
1	1	1.76 × 10^5^	a	Unspoiled	1	2	4.03 × 10^5^	b	Unspoiled
1	2	6.50 × 10^3^	a	Unspoiled	1	3	1.24 × 10^6^	bc	Acceptable
1	3	4.70 × 10^4^	a	Unspoiled	1	4	1.25 × 10^7^	bc	Acceptable
1	4	2.25 × 10^5^	a	Unspoiled	1	7	2.62 × 10^8^	d	Spoiled
1	7	8.36 × 10^6^	b	Acceptable	1	8	2.51 × 10^9^	e	Spoiled
1	8	1.54 × 10^8^	d	Spoiled	1	9	5.71 × 10^9^	f	Spoiled
1	9	2.37 × 10^9^	e	Spoiled	1	10	1.66 × 10^10^	f	Spoiled
1	10	9.20 × 10^9^	e	Spoiled	2	1	4.00 × 10^5^	b	Unspoiled
2	0	1.50 × 10^4^	a	Unspoiled	2	4	3.60 × 10^6^	bc	Acceptable
2	3	2.52 × 10^5^	a	Unspoiled	2	5	7.00 × 10^7^	c	Spoiled
2	4	5.54 × 10^5^	a	Unspoiled	2	6	2.83 × 10^8^	d	Spoiled
2	5	5.27 × 10^6^	b	Acceptable	2	7	1.94 × 10^9^	e	Spoiled
2	6	2.08 × 10^7^	c	Spoiled	2	8	2.60 × 10^9^	e	Spoiled
2	7	1.56 × 10^8^	d	Spoiled	2	11	7.57 × 10^8^	de	Spoiled
2	10	5.15 × 10^9^	e	Spoiled	3	0	3.35 × 10^4^	ab	Unspoiled
3	0	2.00 × 10^5^	a	Unspoiled	3	0	4.67 × 10^3^	a	Unspoiled
3	3	4.22 × 10^6^	b	Acceptable	3	1	1.25 × 10^4^	ab	Unspoiled
3	4	3.30 × 10^6^	b	Acceptable	3	1	2.53 × 10^4^	ab	Unspoiled
3	5	2.04 × 10^8^	d	Spoiled	3	2	2.03 × 10^4^	ab	Unspoiled
* Different letters in each column indicate significant difference at 95% confidence levels as obtained by LSD test.					3	2	2.43 × 10^4^	ab	Unspoiled
					3	3	2.25 × 10^4^	ab	Unspoiled

**Table 5:** One-way ANOVA and LSD test applied on microbiological data (TVC, CFU/g of product) collected on plaice and salmon samples.

EUROPEAN PLAICE					SALMON				
Series	Time (days)	CFU/g	ANOVA LSD *	Group	Series	Time (days)	CFU/g	ANOVA LSD *	Group
1	1	7.20 × 10^6^	b	Acceptable	1	1	6.09 × 10^4^	a	Unspoiled
1	1	1.13 × 10^5^	a	Unspoiled	1	1	8.05 × 10^4^	a	Unspoiled
1	2	1.19 × 10^7^	b	Acceptable	1	2	1.46 × 10^5^	a	Unspoiled
1	2	1.75 × 10^7^	b	Acceptable	1	2	1.10 × 10^5^	a	Unspoiled
1	3	2.07 × 10^8^	e	Spoiled	1	3	1.36 × 10^7^	b	Acceptable
1	3	1.87 × 10^8^	e	Spoiled	1	3	1.10 × 10^7^	b	Acceptable
1	4	1.53 × 10^8^	d	Spoiled	1	4	1.49 × 10^6^	ab	Unspoiled
1	4	1.96 × 10^8^	e	Spoiled	1	4	1.30 × 10^7^	bc	Acceptable
1	5	9.67 × 10^7^	c	Spoiled	1	5	1.85 × 10^7^	bc	Acceptable
1	5	1.24 × 10^8^	c	Spoiled	1	5	2.90 × 10^7^	bc	Acceptable
2	1	3.23 × 10^6^	ab	Unspoiled	1	8	4.15 × 10^8^	d	Spoiled
2	1	2.14 × 10^6^	ab	Unspoiled	1	8	3.41 × 10^8^	d	Spoiled
2	1	1.77 × 10^6^	ab	Unspoiled	2	1	1.56 × 10^5^	a	Unspoiled
2	2	1.18 × 10^7^	b	Acceptable	2	1	1.04 × 10^5^	a	Unspoiled
2	2	1.68 × 10^7^	b	Acceptable	2	2	6.10 × 10^5^	ab	Unspoiled
2	3	2.31 × 10^7^	b	Acceptable	2	2	5.10 × 10^4^	a	Unspoiled
2	3	1.52 × 10^7^	b	Acceptable	2	3	8.52 × 10^5^	ab	Unspoiled
2	4	2.14 × 10^8^	e	Spoiled	2	3	1.17 × 10^6^	ab	Unspoiled
2	4	2.91 × 10^8^	f	Spoiled	2	4	3.40 × 10^7^	c	Acceptable
2	5	1.51 × 10^9^	h	Spoiled	2	4	2.17 × 10^7^	c	Acceptable
2	5	5.25 × 10^8^	g	Spoiled	2	5	1.01 × 10^8^	d	Spoiled
* Different letters in each column indicate significant difference at 95% confidence levels as obtained by LSD test.					2	5	1.23 × 10^8^	d	Spoiled
					2	8	2.69 × 10^9^	e	Spoiled
					2	8	8.57 × 10^8^	de	Spoiled

**Table 6:** Grouping of the analysed samples into three classes.

	BEEF	POULTRY	EUROPEAN PLAICE	SALMON
	(CFU/g)	(CFU/g)	(CFU/g)	(CFU/g)
**Green**	≤10^6^	≤10^6^	≤3 × 10^6^	≤1.5 × 10^6^
**Yellow**	10^6^ < x ≤ 10^7^	10^6^ < x ≤ 1.2 × 10^7^	3 × 10^6^ < x ≤ 5 × 10^7^	1.5 × 10^6^ < x ≤ 5 × 10^7^
**Red**	>10^7^	>1.2 × 10^7^	>5 × 10^7^	>5 × 10^7^

**Table 7:** Results of the K-NN models developed for the e-nose data collected for beef, poultry, European plaice, and salmon. Sensitivity, specificity percentage, and p-values obtained for each class (Unspoiled, US; Acceptable, A; Spoiled, S) in calibration, cross-validation, and prediction of the external test set.

	CALIBRATION			CROSS-VALIDATION			PREDICTION		
Class	US	A	S	US	A	S	US	A	S
**BEEF**									
**Samples**	34	13	8	34	13	8	2	3	20
**Sensitivity**	0.91	0.77	0.75	0.94	0.77	0.75	0.100	0.67	0.95
**Specificity**	0.86	0.88	1.00	0.86	0.90	1.00	0.95	0.95	1.00
***p*-values**	0.91	0.81	0.86	0.91	0.81	0.86	1.00	0.80	1.00
**POULTRY**									
**Samples**	37	10	11	37	10	11	3	2	25
**Sensitivity**	0.89	0.70	0.91	0.92	0.70	0.91	0.66	1.00	0.84
**Specificity**	0.86	0.89	1.00	0.86	0.92	1.00	1.00	0.82	1.00
***p*-values**	0.91	0.78	1.00	0.91	0.78	1.00	0.91	0.90	1.00
**EUROPEAN PLAICE**									
**Samples**	11	14	18	11	14	18	1	7	12
**Sensitivity**	1.00	0.79	0.94	1.00	0.71	0.89	1.00	0.75	1.00
**Specificity**	0.97	0.97	0.92	0.97	0.93	0.88	1.00	1.00	0.78
***p*-values**	0.92	0.92	0.90	0.92	0.91	0.89	1.00	1.00	0.95
**SALMON**									
**Samples**	29	13	5	29	13	5	4	8	13
**Sensitivity**	0.97	0.85	0.80	0.93	0.85	0.80	0.75	1.00	0.92
**Specificity**	0.89	0.94	1.00	0.89	0.91	1.00	1.00	0.88	1.00
***p*-values**	0.93	0.84	1.00	0.93	0.83	1.00	0.96	0.96	1.00

**Table 8:** Results of PLS-DA models developed for the e-nose data collected for beef, poultry, European plaice, and salmon. Sensitivity, specificity percentage, and p-values obtained for each class (Unspoiled, US; Acceptable, A; Spoiled, SP) in calibration, cross-validation, and prediction of the external test set.

	CALIBRATION			CROSS-VALIDATION			PREDICTION		
Class	US	A	S	US	A	S	US	A	S
**BEEF**									
**Samples**	34	13	8	34	13	8	2	3	20
**Sensitivity**	0.82	0.69	0.88	0.82	0.54	0.88	1.00	0.67	1.00
**Specificity**	0.91	0.86	0.94	0.81	0.86	0.94	1.00	1.00	0.80
***p*-values**	0.93	0.80	0.80	0.90	0.80	0.80	1.00	1.00	0.95
**POULTRY**									
**Samples**	37	10	11	37	10	11	3	2	25
**Sensitivity**	0.81	0.80	0.91	0.84	0.70	0.91	0.67	1.00	0.92
**Specificity**	1.00	0.83	0.96	1.00	0.85	0.94	1.00	0.89	1.00
***p*-values**	1.00	0.80	0.76	1.00	0.80	0.76	1.00	0.80	1.00
**EUROPEAN PLAICE**									
**Samples**	11	14	18	11	14	18	1	7	12
**Sensitivity**	1.00	0.93	0.83	1.00	0.79	0.78	1.00	1.00	0.92
**Specificity**	1.00	0.90	0.96	1.00	0.86	0.88	1.00	0.92	1.00
***p*-values**	1.00	0.81	0.93	1.00	0.73	0.82	1.00	0.88	1.00
**SALMON**									
**Samples**	29	13	5	29	13	5	4	8	13
**Sensitivity**	0.97	0.92	1.00	0.97	0.85	0.40	1.00	0.75	100
**Specificity**	1.00	0.97	0.98	1.00	0.88	0.95	1.00	1.00	0.83
***p*-values**	1.00	0.92	0.83	1.00	0.90	0.80	1.00	1.00	0.86

**Table 9:** Prediction results of K-NN and PLS-DA models for beef, poultry, European plaice, and salmon in term of weighted Sensitivity (%) and weighted Specificity (%) and their comparison by McNemar’s test.

	Model	Beef	Poultry	European Plaice	Salmon
**Sensitivity**	KNN	0.92	0.83	0.91	0.92
	PLS-DA	0.96	0.90	0.95	0.92
**Specificity**	KNN	0.97	0.93	0.92	0.96
	PLS-DA	0.84	0.99	0.97	0.91
**E**	KNN	0.12	0.17	0.10	0.08
	PLS-DA	0.04	0.10	0.05	0.08
***p*-values**		0.25	0.25	0.625	1
**H_0_: KNN = PLS-DA**		Equal predictive accuracies	Equal predictive accuracies	Equal predictive accuracies	Equal predictive accuracies
E, classification loss that summarises the accuracy of the classes predicted by k-NN or PLS-DA, *p*-value, *p*-value of the test, H0, Hypothesis test.					

## References

[1] Sen D.P. (2005). Advaced in Fish Processing Technology.

[2] Luning P.A., Jacxsens L., Rovira J., Osés S.M., Uyttendaele M., Marcelis W.J. (2011). A concurrent diagnosis of microbiological food safety output and food safety management system performance: Cases from meat processing industries. Food Control.

[3] Commission Regulation (EC) N On microbiological criteria for foodstuffs. https://www.fsai.ie/uploadedFiles/Reg2073_2005(1).pdf.

[4] Linee Guida per l’analisi del rischio nel campo della microbiologia degli alimenti. https://www.ceirsa.org/docum/allegato_punto4.pdf.

[5] Martinsdottir E., Luten J.B., Schelvis A.A.M., Hydig G., Luten J.B., Oehlenschlager J., Olafsdottir G. (2003). Development of QIM-past and future. Quality of Fish from Catch to Consumers.

[6] Stutz H.K., Silverman G.J., Angelini P., Levin R.E. (1991). Bacteria and Volatile Compounds Associated with Ground Beef Spoilage. J. Food Sci..

[7] Wu L., Pu H., Sun D.W. (2019). Novel techniques for evaluating freshness quality attributes of fish: A review of recent developments. Trends Food Sci. Technol..

[8] ohnson J., Atkin D., Lee K., Sell M., Chandra S. (2019). Determining meat freshness using electrochemistry: Are we ready for the fast and furious?. Meat Sci..

[9] Wilson A.D. (2018). Applications of Electronic-Nose Technologies for Noninvasive Early Detection of Plant, Animal and Human Diseases. Chemosensors.

[10] Loutfi A., Coradeschi S., Mani G.K., Shankar P., Rayappan J.B.B. (2015). Electronic noses for food quality: A review. J. Food Eng..

[11] Deisingh A.K., Stone D.C., Thompson M. (2004). Applications of electronic noses and tongues in food analysis. Int. J. Food Sci. Technol..

[12] Gliszczyńska-Świgło A., Chmielewski J. (2017). Electronic Nose as a Tool for Monitoring the Authenticity of Food. A Review. Food Anal. Methods.

[13] El Barbri N., Llobet E., El Bari N., Correig X., Bouchikhi B. (2008). Electronic nose based on metal oxide semiconductor sensors as an alternative technique for the spoilage classification of red meat. Sensors.

[14] Boothe D.D.H., Arnold J.W. (2002). Electronic nose analysis of volatile compounds from poultry meat samples, fresh and after refrigerated storage. J. Sci. Food Agric..

[15] O’Connell M., Valdora G., Peltzer G., Martín Negri R. (2001). A practical approach for fish freshness determinations using a portable electronic nose. Sens. Actuators B Chem..

[16] Berna A. (2010). Metal oxide sensors for electronic noses and their application to food analysis. Sensors.

[17] Ankara Z., Kammerer T., Gramm A., Schütze A. (2004). Low power virtual sensor array based on a micromachined gas sensor for fast discrimination between H2, CO and relative humidity. Sens. Actuators B Chem..

[18] Ramírez H.L., Soriano A., Gómez S., Iranzo J.U., Briones A.I. (2018). Evaluation of the Food Sniffer electronic nose for assessing the shelf life of fresh pork meat compared to physicochemical measurements of meat quality. Eur. Food Res. Technol..

[19] Ni Y., Kokot S. (2008). Does chemometrics enhance the performance of electroanalysis?. Anal. Chim. Acta.

[20] Cheng J.H., Sun D.W., Zeng X.A., Liu D. (2015). Recent Advances in Methods and Techniques for Freshness Quality Determination and Evaluation of Fish and Fish Fillets: A Review. Crit. Rev. Food Sci. Nutr..

[21] Najam ul Hasan, Ejaz N., Ejaz W., Kim H.S. (2012). Meat and fish freshness inspection system based on odor sensing. Sensors.

[22] Natale C.D., Olafsdottir G., Einarsson S., Martinelli E., Paolesse R., D’Amico A. (2001). Comparison and integration of different electronic noses for freshness evaluation of cod-fish fillets. Sens. Actuators B Chem..

[23] UST Umweltsensortechnik GmbH sensors. http://www.umweltsensortechnik.de/en/gas-sensors/mox-gas-sensors-overview.html.

[24] Brooks J.C., Alvarado M., Stephens T.P., Kellermeier J.D., Tittor A.W., Miller M.F., Brashears M.M. (2016). Spoilage and Safety Characteristics of Ground Beef Packaged in Traditional and Modified AtmospherePackages. J. Food Prot..

[25] (2013). ISO 4833-2: Microbiology of the Food Chain-Horizontal Method for the Enumeration of Microorganisms: Part 1: Colony Count at 30 °C by the Pour Plate Technique.

[26] Wold S., Esbensen K., Geladi P. (1987). Priciple component analysis. Chemom Intell. Lab. Syst..

[27] Wu Y., Ianakiev K., Govindaraju V. (2002). Improved k-nearest neighbor classification. Pattern Recognit..

[28] Ballabio D., Consonni V. (2013). Classification tools in chemistry. Part 1: Linear models. PLS-DA. Anal. Methods.

[29] Kennard R.W., Stone L.A. (1969). Computer Aided Design of Experiments. Technometrics.

[30] Roggo Y., Duponchel L., Huvenne J.P. (2003). Comparison of supervised pattern recognition methods with McNemar’s statistical test: Application to qualitative analysis of sugar beet by near-infrared spectroscopy. Anal. Chim. Acta.

[31] Grassi S., Casiraghi E., Alamprese C. (2018). Handheld NIR device: A non-targeted approach to assess authenticity of fish fillets and patties. Food Chem..

[32] Panigrahi S., Balasubramanian S., Gu H., Logue C.M., Marchello M. (2006). Design and development of a metal oxide based electronic nose for spoilage classification of beef. Sens. Actuators B Chem..

[33] Panigrahi S., Balasubramanian S., Gu H., Logue C., Marchello M. (2006). Neural-network-integrated electronic nose system for identification of spoiled beef. LWT-Food Sci. Technol..

[34] Olafsdottir G., Lauzon H.L., Martinsdóttir E., Oehlenschläger J., Kristbergsson K. (2006). Evaluation of shelf life of superchilled cod (Gadus morhua) fillets and the influence of temperature fluctuations during storage on microbial and chemical quality indicators. J. Food Sci..

